# Data on transcriptome profiling of circulating microRNAs in dairy cattle

**DOI:** 10.1016/j.dib.2018.09.038

**Published:** 2018-09-18

**Authors:** Kesavan Markkandan, Hyun-joo Lim, Dong Jin Lee, In-gang Shin, Seung-il Yoo, Changgwon Dang, Chang Pyo Hong, Kung Ahn

**Affiliations:** a Theragen ETEX Bio Institute, Suwon, Gyeonggi-do 16229, Republic of Korea; b National Institute of Animal Science (NIAS), RDA, Cheonan 331-801, Republic of Korea

**Keywords:** Circulating microRNA, Holstein, MiRNA-sequencing, Exosome, Pregnancy

## Abstract

MicroRNA (miRNA) are found in numerous biofluids including blood and are considered a new class of biomarkers. The data presented here are related to the research article entitled “Profiling and identification of pregnancy-associated circulating microRNAs in dairy cattle” (Markkandan et al. 2018). In the cited article, we sequenced the circulating microRNAs of the three healthy dairy cows of normal and 30 days of pregnancy (DOP) using Illumina RNA-Seq. Differentially expressed genes (DEG) analysis between normal and pregnant samples showed perturbations in miRNA expression. Herein, we made a comparison of DEGs at normal and 60 DOP libraries. The analysis results showed that 147 known miRNAs were differently expressed at 60 DOP groups when compared to the normal group. In addition, stage specific miRNAs were also predicted.

**Specifications table**

**Table:** 

Subject area	Biology
More specific subject area	Transcriptomics
Type of data	Table, figure
How data was acquired	Illumina HiSeq. 2500
Data format	Raw (FASTQ)
Experimental factors	Blood samples of normal and 60 days of pregnancy were collected from three Holstein dairy cattle
Experimental features	DEGs of normal and 60 days of pregnancy
Sample source location	National Institute of Animal Science (NIAS), Cheonan, Republic of Korea
Data accessibility	Data is available in the article and at: https://www.ncbi.nlm.nih.gov/Traces/study/?acc=SRP151837

**Value of the data**

• The differentially expressed miRNAs could serve as potential biomarkers for not only early diagnosis of pregnancy but also for livestock health and disease.
• Pregnancy-associated microRNA profiling at 60 DOP in bovine was described for the first time and can be used for comparative studies.
• These miRNAs may have similar function in mammalian species and can be potential molecular markers for evolution.

## 1 Data

**Fig. 1:**
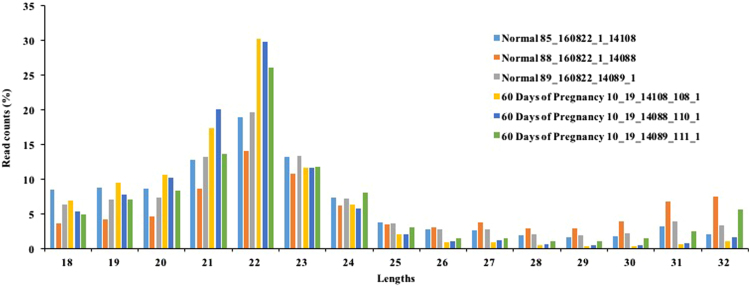
Length distribution of miRNA sequences in normal and 60 days of pregnancy samples by Illumina small RNA deep sequencing. Sequence length distribution of clean reads based on the abundance and distinct sequences; the most abundant size class was 22 nt, followed by 21 and 23 nt.

Data shown in this article are related to the research article entitled “Profiling and identification of pregnancy-associated circulating microRNAs in dairy cattle” [1]. The exosomal miRNA extracted from the serum blood of normal and 60 DOP samples were analyzed. The quality of sequencing reads was assessed by the read length (Fig. 1, Supplementary Table 1). Table 1 describes the number of differentially expressed miRNAs at 60 days of pregnant cows. The target gene prediction for those differentially expressed miRNAs revealed that, 21,801 genes were regulated by 147 differentially expressed miRNAs (Supplementary Table 2).

**Table 1:** Differential miRNA profile expression between normal and 60 days of pregnancy groups (*p*-value < 0.05).

Differentially expressed miRNAs	60 days of pregnancy groups (This study)	30 days of pregnancy groups [1]
Up	88	21
Down	59	8

## 2 Experimental design, materials and methods

### 2.1 Animals and collection of samples

The whole blood samples of before and 60 DOP were collected from three individual healthy Holstein cow. Briefly, about 10 mL whole blood was collected in EDTA tubes, and then the samples were incubated at room temperature for 1 h. The whole blood samples were centrifuged at 4 °C (1900×*g*, 10 min), followed by a second centrifugation at 4 °C (16,000×*g*, 10 min). After this step, the circulating cell-free nucleic acid were in the supernatant (sera) and then the sera were transferred into a fresh 2.0 mL tube and stored at −80 °C for further analysis.

### 2.2 Extraction total RNA, Illumina sequencing and data analysis

Before RNA isolation, the exosomes in sera was firstly isolated using Total Exosomes Isolation kit (Invitrogen, Carlsbad, CA). Exosomal total RNA was isolated using miRNeasy Mini Kit (Qiagen) following manufacturer׳s protocols. The concentration of RNA was determined using the Qubit microRNA assay kit (Invitrogen, Carlsbad, CA). The RNA samples were stored at −80 °C for further analysis. Total RNA (5 µL) from each sample was used to construct miRNA library using the NEXTflex Small RNA Sequencing Kit (Illumina, San Diego, CA) according to the manufacturer׳s instruction. Small RNA libraries were then pooled together in equal volumes for gel purification. The pooled library was sequenced using the HiSeq. 2500 system (Illumina) as 50 bp single reads. Read quality (adaptor removal, and size selection) was assessed using FastQC v0.11.5 (http://www.bioinformatics.babraham.ac.uk/projects/fastqc/) and cutadapt [2]. The sequences with read length larger than 18 nucleotides (nt) were aligned against bovine miRNA database (miRBase, release version 21) with the default parameters to identify known miRNAs using Bowtie2 [3]. Each library was processed separately. The expression level of miRNAs in each library was estimated by sRNAbench, which normalized reads count number of each miRNA reads per million (RPM) by the following formula: RPM=(miRNA reads number/total mapped reads per library)×1,000,000. The DE miRNAs were determined by Log^2^ fold change (FC) >1 or <−1 and false discovery rate (FDR) < 0.05 based on counting reads using HTSeq [4]. Briefly, target genes of selected miRNAs were commonly predicted by TargetScan [5] and miRanda (http://www.microrna.org/microrna/home.do).

## Appendix A Supplementary material

Supplementary Table 1. Sequencing and miRNA profiling statistics of normal and 60 days of pregnancy groups.

.

Supplementary Table 2. The number of predicted target genes.
